# Progress in controlling the transmission of schistosome parasites in Southern Ethiopia: the Geshiyaro Project in the Wolaita Zone

**DOI:** 10.1186/s13071-024-06156-1

**Published:** 2024-03-06

**Authors:** Birhan Mengistu, Ewnetu Firdawek Liyew, Melkie Chernet, Geremew Tasew, Santiago Rayment Gomez, Rosie Maddren, Benjamin Collyer, Ufaysa Anjulo, Adugna Tamiru, Kathryn Forbes, Zelalem Mehari, Kebede Deribe, Teshale Yadeta, Mihretab Salasibew, Getachew Tollera, Roy Anderson

**Affiliations:** 1 London Centre for Neglected Tropical Disease Research, Department of Infectious Disease Epidemiology, Faculty of Medicine, St Marys Campus, Imperial College London, London, UK; 2 Bacterial, Parasitic and Zoonotic Disease Research Directorate, Ethiopian Public Health Institute, Addis Ababa, Ethiopia; 3 Disease Prevention and Health Promotion Core Process, Ministry of Health, Addis Ababa, Ethiopia; 4 Children’s Investment Fund Foundation, London, UK

**Keywords:** Schistosomiasis (SCH), Mass drug administration (MDA), Kato-Katz (KK), Point-of-care circulating cathodic antigen (POC-CCA)

## Abstract

**Background:**

This paper describes changes in the prevalence and intensity of schistosome parasite infections in a project integrating mass drug administration (MDA), water, sanitation, and hygiene (WaSH), and behavioral change interventions.

**Methods:**

The Geshiyaro Project comprises three intervention arms. Arm 1 is subdivided into “Arm 1 pilot” (one district) and Arm 1 (four other districts), both receiving integrated community-wide MDA with intensive WaSH interventions. Arm 2 involves 17 districts with community-wide MDA interventions, while Arm 3 serves as a control with school-based MDA interventions in three districts. A total of 150 individuals, stratified by age group, were randomly selected from each of the 45 sentinel sites. Arm sizes were 584 (Arm 1 pilot), 1636 (Arm 1), 2203 (Arm 2), and 2238 (Arm 3). Statistical tests were employed to compare infection prevalence and intensity across the different arms.

**Results:**

The prevalence of schistosome parasite infection ranged from 0% to 2.6% and from 1.7% to 25.7% across districts, employing the Kato-Katz (KK) and point-of-care circulating cathodic antigen (POC-CCA) diagnostics, respectively. The mean infection intensity level showed no marked difference between baseline and follow-up surveys when measured by KK, except in Arm 2 (*t* = 6.89, *P* < 0.0001). Infection prevalence decreased significantly in Arm 1 (*t* = 8.62, *P* < 0.0001), Arm 2 (*t* = 6.94, *P* < 0.0001), and Arm 3 (*t* = 8.83, *P* < 0.0001), but not in Arm 1 pilot (*t* = 1.69, *P* = 0.09) by POC-CCA, when trace was considered positive. The decrease was significant only in Arm 1 (*t* = 3.28, *P* = 0.0001) and Arm 2 (*t* = 7.62, *P* < 0.0001) when the trace was considered negative in POC-CCA. Arm 2 demonstrated a significant difference in difference (DID) compared to the control group, Arm 3, regardless of whether trace in POC-CCA was considered positive (DID = 3.9%, *df* = 8780, *P* = 0.025) or negative (DID = −5.2, *df* = 8780, *P* = 0.0004).

**Conclusions:**

The prevalence of schistosomiasis was low when employing the KK diagnostic but moderate in some locations by the POC-CCA diagnostic. The infection level had decreased across all arms of the Geshiyaro study at mid-term of the 7-year project, but further efforts are needed to reduce the rate of parasite transmission based on the POC-CCA diagnostic scores.

**Graphical Abstract:**

**Figure:**
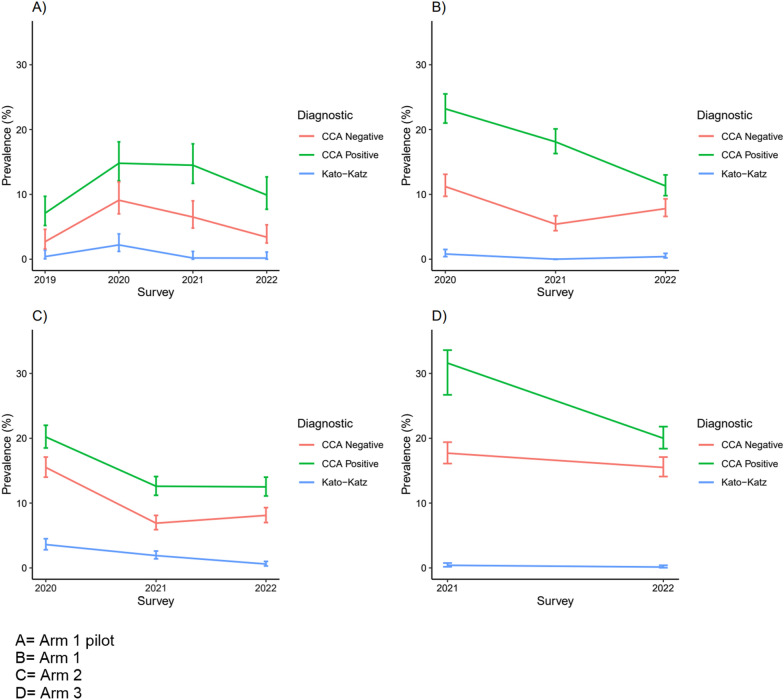

**Supplementary Information:**

The online version contains supplementary material available at 10.1186/s13071-024-06156-1.

## Background

Schistosomiasis (SCH) is one of the 20 neglected tropical diseases (NTDs) defined by the World Health Organization (WHO). It is known to cause cognitive and developmental problems, especially in children. Infection with two parasite species, *Schistosoma mansoni* and *Schistosoma haematobium*, is the dominant cause of the disease in sub-Saharan Africa. Transmission occurs when the parasite’s larval stage, the cercaria, penetrates a person’s skin on exposure to an infected water body. WHO estimated in 2016 that more than 240 million people worldwide were infected with schistosome parasites, resulting in 2.5 million disability-adjusted life years (DALY) and 24,000 deaths. Most infections occur in Africa, and most people requiring SCH treatment live on the same continent [1, 2]. SCH is common in low-income communities with limited access to clean water and poor sanitation facilities [3].

Mass drug administration (MDA) using praziquantel to all eligible people in endemic areas regardless of their infection status is the main strategy for the control and elimination of SCH. This is termed prophylactic MDA. The new WHO guideline released in 2022 suggests treating all age groups above 2 years of age, including pregnant women after the first trimester and lactating mothers, in areas that have a prevalence of 10% and above, in order to control and eliminate morbidity caused by infection and lead the way to transmission interruption [4]. Pediatric praziquantel has recently been through clinical trials and has shown promise in treating children less than 6 years of age [5]. This new drug formulation could enable the treatment and control of schistosome infection among preschool-age children.

Ideally, SCH control and the elimination of parasite transmission requires a combination of MDA and improved access to clean water, sanitation, and good hygiene practice (WaSH). Environmental management to limit the growth of the snail intermediate host and the implementation of health education interventions are also desirable [1]. Elimination of SCH and maintaining a low prevalence level is challenging using MDA as the only control strategy, unless MDA population coverage is very high [6, 7].

The progress achieved over the past decade through MDA control could be reversed if other interventions like WaSH are not implemented to secure longer-term reductions in the likelihood of persistent transmission, as decreased drug donations or supply problems limit MDA coverage [8, 9].

Intestinal SCH (caused by *S. mansoni*) and urinary SCH (caused by *S. haematobium*) are major public health problems in Ethiopia. The first *S. mansoni* case in Ethiopia was recorded in 1934, and *S. haematobium* was recorded later in a few localities in the Rift Valley [10]. In 1964, the first cases of *S. mansoni* associated with an introduced irrigation system were found in laborers in the Wonji sugar estate 10 years after the system was built. Infection prevalence was 20% in 1980 among local communities and school [11]. In 1961, SCH was reported in the highland (commonly *S. mansoni*) and lowland areas (commonly *S. haematobium*) of the country [11]. According to surveys conducted between 1979 and1982, the prevalence of *S. mansoni* was found to be around 14%. The control of SCH in Ethiopia used to be very local, reliant on specific project-based funding by a variety of non-governmental organizations (NGOs), and usually concentrated on the small-scale distribution of praziquantel and reduction of snail populations through molluscicide application (using a local herb called endod). The prevalence decreased from 64% to 33% in one locality after 5 years of implementation. However, the endemic foci for SCH increased from 8 to 55 between 1960 and 2011 due to population movement and ecological changes that have created suitable environments for snails and concomitantly parasite transmission [12]. The application of endod as a molluscicide was hampered by its cost and scarcity [11].

The first nationwide SCH mapping program integrated with soil-transmitted helminthiasis (STH) was conducted between 2013 and 2015. The dominant species was found to be *S. mansoni* (3.5% vs. 0.5% *S. haematobium*) using Kato Katz (KK) and urine filtration methods, respectively. There was significant heterogeneity across regions in levels of infection as recorded by population prevalence. Benishangul-Gumuz (14.9%) had the highest prevalence, followed by Tigray (10.8%) and Gambella (9.2). A total of 385 districts (Woreda) were identified with infection and hence requiring MDA [13, 14].

A national deworming program was launched in 2015 with the aim of eliminating SCH as a public health problem in Ethiopia. School-age children (SAC) and adults in high-risk districts were treated according to WHO guidelines [15]. The prevalence of heavy infections decreased by 65% through the treatment of more than 27 million people over 5 years. However, SCH remains a public health problem with pockets of infection in many areas, and effective control requires more intensive intervention, as the country aims for the interruption of transmission by 2030 [16].

Geshiyaro is a pilot operational research project funded by the Children’s Investment Fund Foundation (CIFF) and is being implemented in partnership with the Ministry of Health in collaboration with World Vision Ethiopia (WVE). The project, which is in its fifth year of implementation, aims to test the feasibility of interruption of SCH and STH by integrating MDA, WaSH, and behavioral interventions. The project is not a clinical trial, but an enhanced control program administered within the existing Ministry of Health NTD control activities, with MDA delivered by local health extension workers. The protocol of the project is described in detail in a separate publication [17]. Planned progress in the project and the implementation of MDA activities were greatly influenced by the SARS-CoV-2 pandemic in the years 2020–2022.

This paper describes the changes in the prevalence and intensity of SCH within the Geshiyaro Project in the Wolaita Zone of Southern Ethiopia measured by both the KK and point-of-care circulating cathodic antigen (POC-CCA) diagnostic tests. The progress in STH control has been published elsewhere (Birhan et. al; 2023 in press).

## Methods

### Study area

The study area is the Wolaita Zone of the Southern Nations, Nationalities, and Peoples’ Region (SNNPR) of Ethiopia. Sodo town is the zonal administrative hub, which is 330 km south of Addis Abeba, the capital city of Ethiopia. The Wolaita Zone has large borders with the Sidama regions. It also shares smaller borders with the Hadiya, Gamo, Gofa, Kembata-Tembaro and Dawro zones. There are 22 districts in the Wolaita Zone (increased from 15 at baseline due to the redefinition of district boundaries) grouped in either Arm 1 of the study, which employs community-wide MDA and expanded WaSH, or Arm 2, which employs community-wide MDA and the existing government WaSH program [17]. In Arm 3, school-based MDA and the existing government WaSH program are employed (i.e., no additional control activity over and above the existing government NTD control program). There are three districts in Arm 3 located in the Oromia and Sidama regions. There are two rivers (Bilate and Omo) and two lakes (Abaya and Chamo) in and around Wolaita. Hawassa lake and Werka and Wosho rivers are found in Arm 3 districts [18, 19]. The zone had an estimated population of 2.2 million people in 2022 [20]. The size of the zone is about 4511 km^2^, with the lowest point at Bilate (501 m) and the highest point at Mount Damota (2958 m). The average annual temperature ranges from a low of 15.2 °C to a high of 31.4 °C, and annual rainfall is between 800 and 1600 mm [21].

### Study design

The Geshiyaro Project implemented a combination of community-wide MDA, WaSH, and behavioral change interventions in Arm 1. In Arm 2, there were community-wide MDA interventions and one WaSH (government-led) intervention. Arm 3 involved a school-based MDA treatment and the existing WaSH government program. The project was initiated in 2018/2019 in Boloso Sore district (referred to as Arm 1 pilot in this paper) and later expanded to four additional districts under Arm 1, referred to as Arm 1. Arm 1 was subdivided into two groups to account for the difference in implementation periods at different times due to the impact of the coronavirus disease 2019 (COVID-19) pandemic. Seventeen districts were included in Arm 2. There were three more districts in Arm 3 which were designated as the control group in the sense that it included no additional control activities over and above the existing government-run interventions (school-based MDA and existing WaSH program).

The Geshiyaro Project’s research framework involves the establishment of annual longitudinal sentinel sites [17]. These sites serve as cohorts to investigate trends in prevalence and intensity reduction, explore the association between infection and treatment/coverage, and examine the relationship between infection and access to WaSH facilities. The project outlined a sample of 150 individuals per site, with a total of 45 sites (15 from districts in each arm). The design includes monitoring a broader range of age classes, including adults and preschool-age children, in addition to SAC. The sentinel sites were selected based on baseline mapping and stratification by co-endemicity, ensuring a balance of sites across different infection categories. Recruitment for sentinel sites was conducted door-to-door, with consent obtained for study participation.

This study examines data collected from a series of sentinel sites where cohorts of people are monitored for infection status (prevalence and intensity) longitudinally, in which the prevalence and intensity of SCH and STH infection are examined each year of the study within a defined sample of people stratified by age and gender using a sampling frame as described in the protocol [17] to record the progress in control. The prevalence and intensity of SCH were determined annually from the 45 longitudinal sentinel survey sites, with 150 people sampled from each. In this paper, baseline and three follow-up (FU) survey results were included from the Arm 1 pilot. The baseline and two follow-up survey results were analyzed in the rest of Arm 1 and Arm 2. However, only the baseline and one follow-up survey result were considered in Arm 3. COVID-19 affected the timing of the interventions in the different arms of the study. The timeline for baseline and follow-up surveys is shown in Table 1, and the protocol for this study was published elsewhere [17].

**Table 1:** Parasitological survey and MDA implementation by arm and year in the Geshiyaro Project

Arm	District name	Baseline	Follow-up survey 1 (FU1)	Follow-up survey 2 (FU2)	Follow-up survey 3 (FU3)	Round 1 MDA	Round 2	Round 3 MDA
Arm 1 pilot	Boloso Sore	Nov. 2018	Oct. 2019	Oct. 2020	Nov. 2021	Jan. 2019	Nov. 2019	Nov. 2020
Arm 1	Bolosso Bombe, Damot Gale, Damot Pulasa, Damot Sore	Oct. 2020	Nov. 2021	Apr. 2022		Nov. 2020	Dec. 2021	
Arm 2	Damot Weydie Abala Abeya, Araka town, Bayra koisha, Boditi town, Dugna Fango, Gesuba town, Genuno town, Hobicha, Humbo, Kawo Koisha, Kindo Didaye, Kindo Koisha, Ofa, Sodo town, Sodo Zuria	Oct. 2020	Nov. 2021	Apr. 2022		Nov. 2020	Dec. 2021	
Arm 3	Wondo, Wondo Genet, Tula	Mar. 2020	Apr. 2022				Dec. 2021	

### Parasitology

Participants in the longitudinal cohort were asked to provide stool and urine samples in labeled cups. The stool was tested for the presence of *S. mansoni* and intensity using the KK diagnostic method [22]. Four thick smears from two consecutive days of stool samples were prepared. The result was read by trained laboratory technologists employing a light microscope. In addition to KK, the POC-CCA technique was used to determine infection with *S. mansoni* using a urine sample. The POC-CCA results were recorded as trace, 1+, 2+, and 3+. The prevalence of schistosome infections was considered by including trace CCA as either positive or negative. The POC-CCA tests were conducted on all urine samples following the manufacturer’s instructions (supplied by Rapid Medical Diagnostics, South Africa; batch numbers 200326039 for 2019, 210607058 for 2020, 21110105 for 2021, and 230116005 for 2022). The urine was screened for *S. haematobium* using the dipstick technique to detect hematuria and the urinary egg filtration method. The filtration test was done only on urine samples that were positive for hematuria on the dipstick test. The number of eggs per gram of stool and 10 ml of urine was calculated and categorized according to the WHO intensity classification [23]. Participants’ biometric fingerprints were recorded and linked to demographic data collected via smartphones. The data were stored using Survey CTO software (Dobility, Inc., Cambridge, MA, USA). The detailed procedure is published in the Geshiyaro protocol paper [17].

### Data analysis

Data collected in Survey CTO were downloaded in Microsoft Excel format, cleaned for duplicate or missing data entries, and analyzed in RStudio version 4.3.0. (2023-04-21 UCRT). Prevalence and intensity were calculated for the different arms of the study and further stratified by district, age, and gender. Chi-square tests for independence were used to show an association between infection and gender or infection and age. The *t*-test was used to test the significant difference of prevalence at baseline and follow-up surveys employing log-transformed data on intensity given the underlying negative binomial distribution of egg counts with a high variance-to-mean ratio. A chi-square test was conducted to assess whether there was significant difference in prevalence among different age groups. The significance threshold was set at *P* = 0.05. The intensity of infection was calculated and presented for all arms of the study, but no statistical test was performed due to the small number of infected individuals. Tests of significance for trends in disease reduction were performed using non-parametric tests. The difference in difference (DID) was calculated using the following formula: (prevalence of infection at follow-up in intervention group − prevalence of infection at baseline in intervention group)/(prevalence of infection at follow-up in control group − prevalence of infection at baseline in the follow-up group). The DID was calculated for prevalence measured by POC-CCA, but because the prevalence by KK was very small, DID was not determined due to very small sample size.

### Ethical approval

Ethical approval was obtained from the Institutional Review Board (IRB) at the Scientific and Ethical Review Office of the Ethiopian Public Health Institute. A letter of consent was given to all concerned government bodies. Oral consent was sought from participants during each longitudinal survey. Consent was sought for children from their parents or guardian. Participants could choose to join or not without affecting their benefit.

## Results

### Demographic information and sample size

The number of people enrolled in the longitudinal cohort at the baseline and the follow-up surveys, stratified by study arm, age group, and gender, is presented in Additional file 1: Table S1. In the Arm 1 pilot district, there were 568 participants enrolled at baseline in 2018, and 587, 553, and 584 people at follow-up 1 (FU1), follow-up 2 (FU2), and follow-up 3 (FU3) in 2019, 2020, and 2021, respectively. In Arm 1 and Arm 2, data were collected from 1395 and 2161 participants, respectively, in 2020, and 1636 and 2203 participants at baseline and FU2 in 2022, respectively. In Arm 3, 2177 and 2238 participants were engaged at baseline and follow-up 1 in 2020 and 2022, respectively. The participants in sentinel sites were consistently tracked and followed; in cases where they could not be located, individuals with similar demographic characteristics were substituted, and such changes were logged in the cloud-based database using Survey CTO software.

### Impact of interventions on the prevalence of *Schistosoma* infections

In all the arms, the prevalence of SCH and the change in time, as measured by the KK diagnostic, remained notably low (0.4% in 2019 to 0.2% in 2022 for Arm 1 pilot, 0.8% in 2020 to 0.4% in 2022 for Arm 1, 3.6% in 2020 to 0.6% in 2022 for Arm 2, 0.4% in 2021 to 0.1% in 2022 for Arm 3). The prevalence measured by KK showed no significant change over time, except in Arm 2 (*t* = 6.89, *P* < 0.0001).

However, when the POC-CCA test was employed, considering all trace positive results as positive, infection prevalence decreased significantly in Arm 1 (from 23.2% in 2020 to 11.3% in 2022, *t* = 8.62, *P* < 0.0001), Arm 2 (from 20.2% in 2020 to 12.5% in 2022, *t* = 6.94, *P* < 0.0001), and Arm 3 (from 31.6% in 2021 to 20% in 2022, *t* = 8.83, *P* < 0.0001 for all) but not in Arm 1 pilot (from 7.1% in 2019 to 9.9% in 2022, *t* = −1.69, *P* = 0.09). When the POC-CCA data were used with the trace considered negative, a significant decrease in prevalence was observed only in Arm 1 (from 11.2% in 2020 to 7.8% in 2022, *t* = 3.28, *P* = 0.001) and Arm 2 (15.5% in 2020 to 8.1% in 2022, *t* = 7.62, *P* < 0.0001).

There was no notable disparity in infection based on gender across arms, whether KK or POC-CCA was employed. Regarding *S. mansoni* infection measured by POC-CCA, with trace considered positive, the infection levels were slightly elevated in the age group 5–14 years within Arm 1 (*χ*^2^ = 10.62, *df* = 4, *P* = 0.03) when compared with other age groups.

The assessment of *S. mansoni* using POC-CCA exhibited dynamic changes. In the Arm 1 pilot, the trace category increased from 4.4% (2.9–6.6%) at baseline to 6.5% (4.7–8.9%) at FU3, with concurrent increases observed in the 1+, 2+, and 3+ categories (Additional file 2: Table S2). Conversely, both Arm 1 and Arm 2 exhibited decreasing trends across all trace, 1+, 2+, and 3+ categories. Specifically, the trace category decreased substantially from 11.9% (10.3–13.8%) to 3.5% (2.6–4.4%) at FU2. Arm 3 also experienced a significant decrease in the trace category from 13.9% (12.5–15.4%) to 4.5% (3.7–5.5%) at FU1. Notably, in Arm 3, the 3+ category showed an increasing trend at FU1 (Additional file 2: Table S2).

The prevalence of urinary SCH (*S. haematobium*), measured by urinary egg microscopy, was extremely low throughout all surveys, with insufficient data to conduct statistical analyses (Table 2).

**Table 2:** SCH prevalence reduction in all arms by KK and CCA (when trace was considered positive [CCA+] or negative [CCA−])

Arm	*S. mansoni* prevalence (% and 95% CI)							*S. haematobium prevalence* (%)
	Survey	KK	*P*-value	POC-CCA+	*P*-value	POC-CCA−	*P*-value	Urine filtration
Arm 1 pilot	Baseline	0.4 (0.06,1.4)	0.54	7.1 (5.2, 9.7)	0.09*	2.7 (1.6, 4.6)	0.5	0
	FU1	2.7 (1.6, 4.6)		14.8 (12.1, 18.1)		9.1 (7, 11.9)		0
	FU2	0.18 (0, 1.2)		14.5 (11.7, 17.8)		6.5 (4.8, 9)		0
	FU3	0.17 (0,008, 1.1)		9.9 (7.7, 12.7)		3.4 (2.5, 5.3)		0.3
Arm 1^a^	Baseline	0.8 (0.4, 1.5)	0.2	23.2 (21, 25.5)	< 0.0001*	11.2 (9.7, 13.1)	< 0.0001*	0.1
	FU1	0		18.1 (16.3, 20.1)		5.4 (4.4, 6.7)		0
	FU2	0.4 (0.2, 0.9)		11.3 (9.8, 13)		7.8 (6.6, 9.3)		0.06
Arm 2^b^	Baseline	3.6 (2.8, 4.5)	< 0.0001*	20.2 (18.5, 22)	< 0.0001*	15.5 (14, 17.1)	< 0.0001*	0
	FU1	1.9 (1.4, 2.6)		12.6 (11.2, 14.1)		6.9 (5.9, 8.1)		0
	FU2	0.6 (0.3, 1)		12.5 (11.1, 14)		8.1 (7, 9.3)		0
Arm 3^c^	Baseline	0.4 (0.17, 0.75)	0.12	31.6 (26.7, 33.6)	< 0.0001*	17.7 (16.1, 19.4)	0.05	0.09
	FU1	0.13 (0.03, 0.4)		20 (18.4, 21.8)		15.5 (14.1, 17.1)		0

*Statistically significant; *CI* confidence interval; *BL* baseline; *FU* follow-up; *CCA+* cathodic circulating antigen when trace considered positive; *CCA−* cathodic circulating antigen when trace considered negative

^a^In Arm 1 pilot: FU1 was in 2019, FU2 was in 2020, FU3 was in 2021, and FU4 was in 2022

^b^In Arm 1 and Arm 2: FU1 was in 2020, FU2 was in 2021, and FU2 was in 2022

^c^In Arm 3: FU1 was in 2021, and FU2 was in 2022

The DID analysis yielded significant results for Arm 2 compared to Arm 3, regardless of whether trace was considered positive (DID = 3.9%, *df* = 8780, *P* = 0.025) or negative (DID = −5.2, *df* = 8780, *P* = 0.0004), indicating a significant difference in prevalence at baseline in the two arms, with the control group showing higher prevalence. When comparing Arm 1 pilot to Arm 3, a significant DID of 14.4% (*df* = 5541, *P* < 0.0001) was observed when trace was considered positive, suggesting an increase of prevalence from the baseline in the intervention group. However, there was no significant DID (DID=2.9%, *df* = 5541, *P* = 0.06) when trace was considered negative. In comparison between Arm 1 and Arm 3, there was no significant DID when trace was considered either positive or negative.

### Trends in *Schistosoma* infection over time

**Fig. 1:**
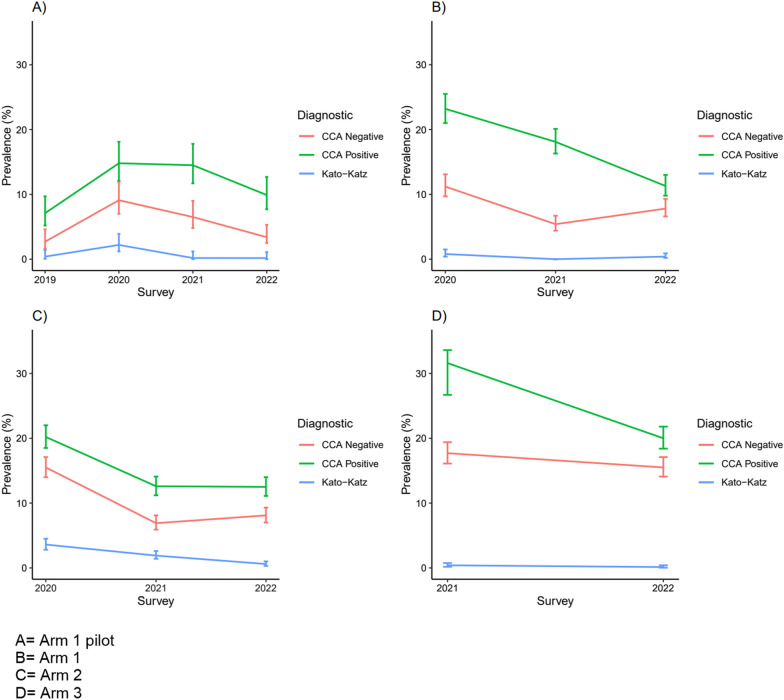
Trends in *S. mansoni* infection, with confidence intervals, in different arms by KK and CCA (when trace was considered positive or negative). **A** Trend in *S. mansoni* infection in Arm 1 pilot; **B** trend in *S. mansoni* infection in Arm 1; **C** trend in *S. mansoni* infection in Arm 2; **D** trend in *S. mansoni* infection in Arm 3

*Schistosoma mansoni* prevalence in the Arm 1 pilot district increased from baseline during FU1 and FU2, then decreased to the baseline level by KK and CCA in FU3 (Fig. 1). A non-parametric trend test was not significant either by KK (*z* = 2, −0.72, *Z* = 0, *P* = 1) from baseline to FU3. In Arm 1, the prevalence, measured by POC-CCA (trace considered negative) was lower at FU1 compared to baseline but then increased on FU2 when the trace was considered negative. However, the prevalence remained the same by KK in the follow-up surveys. In Arm 1, a non-significant declining trend was observed by POC-CCA when trace was considered negative (*z* = 1.045, *P* = 0.29) from baseline to FU2. In Arm 2, the infection was reduced on the FU1 survey and did not change until the FU2 survey using POC-CCA when the trace was considered either positive or negative. A tendency of decreasing trend was seen by KK which was not significant (*z* = −1.04, *P* = 0.29). In Arm 3, the infection had decreased on the FU1 survey when trace was considered either positive or negative. In Arm 3, the infection prevalence continued to be low by KK in the follow-up survey. There are only two surveys in Arm 3, which implies that no trend over time analysis was possible.

### Intensity of infection and egg counts

The intensity of infection for all tested participants measured using KK at the baseline survey in all the arms was light (0.4%, 0.78%, 3.3%, and 0.27% for Arm 1 pilot, Arm 1, Arm 2, and Arm 3, respectively). There was only one case of heavy-intensity infection in Arm 3. In the latest follow-up surveys, *S. mansoni* infections were light, at 0%, 0.3%, 0.6%, and 0.13% in Arm 1 pilot, Arm 1, Arm 2, and Arm 3, respectively. Moderate infection cases were found only in Arm 1 and Arm 2 (Table 3).

**Table 3:** Intensity of *S. mansoni* infection in the different arms at the baseline and follow-up surveys based on the KK diagnostic

	Infected number	Baseline prevalence (%) and 95% CI	Infected number	Follow-up 1 prevalence (%) and 95% CI	Infected number	Follow-up 2 prevalence (%) and 95% CI	Infected number	Follow-up 3 prevalence (%) and 95% CI
Arm 1 pilot								
Light	2	0.4 (0.06, 1.4)	12	2 (1.1, 3.6)	1	0.18 (0.009, 1.5)	0	0
Moderate	0	0	1	0.17 (0.008, 1.1	0	0	1	0.17 (0.008, 1.1)
Heavy	0	0	0	0	0	0	0	0
Arm 1								
Light	11	0.78 (0.04, 1.4)	0	0	5	0.3 (0.1, 0.7)		
Moderate	0	0	0	0	2	0.1 (0.02, 0.5)		
Heavy	0	0	0	0	0	0		
Arm 2								
Light	72	3.3 (2.6, 4.2)	38	1.8 (1.3, 2.5)	13	0.6 (0.3, 1)		
Moderate	5	0.2 (0.08, 0.6)	3	0.14 (0.03, 0.45)	0	0		
Heavy	0	0	0	0	0	0		
Arm 3								
Light	6	0.27 (0.1, 0.6)	3	0.13 (0.03, 0.4)				
Moderate	1	0.05 (0.002, 0.3)	0	0				
Heavy	1	0.05 (0.002, 0.4)	0	0				

*CI* confidence interval

The egg count ranged within specific intervals for different arms and time points. In Arm 1 pilot, the egg count varied from 0 to 72 at baseline and 0 to 300 at follow-up 3. For Arm 1, it spanned from 0 to 48 at baseline and 0 to 306 at follow-up 2. In Arm 2, the range was from 0 to 162 at baseline and 0 to 72 at follow-up 2. Lastly, in Arm 3, the egg count ranged from 0 to 1320 at baseline and 0 to 90 at follow-up 1.

The average egg count shown in Table 4 decreased in Arm 2 from 1.4 to 0.26 eggs per gram (*t* = 5.17, *P* < 0.0001), but no significant change occurred in any other arms.

**Table 4:** Average egg count in different arms at the baseline and follow-up surveys

	Baseline mean egg count per gram (95% CI)	Follow-up 1 mean egg count per gram (95% CI)	Follow-up 2 mean egg count per gram (95% CI)	Follow-up 3 mean egg count per gram (95% CI)	*P*-value
Arm 1 pilot	0.13 (0.01, 0.47)	0.97 (0.36, 1.87)	0.09 (0.01, 0.32)	0.51 (0.01, 1.89)	0.47
Arm 1	0.13 (0.05, 0.26)	0	0.37 (0.09, 0.87)		0.25
Arm 2	1.4 (1.01, 1.80)	0.93 (0.63, 1.30)	0.26 (0.14, 0.44)		< 0.0001*
Arm 3	0.78 (0.06, 2.36)	0.07 (0.01, 0.18)			0.24

*Statistically significant. *CI* confidence interval

### Prevalence of schistosome infection by district

**Fig. 2:**
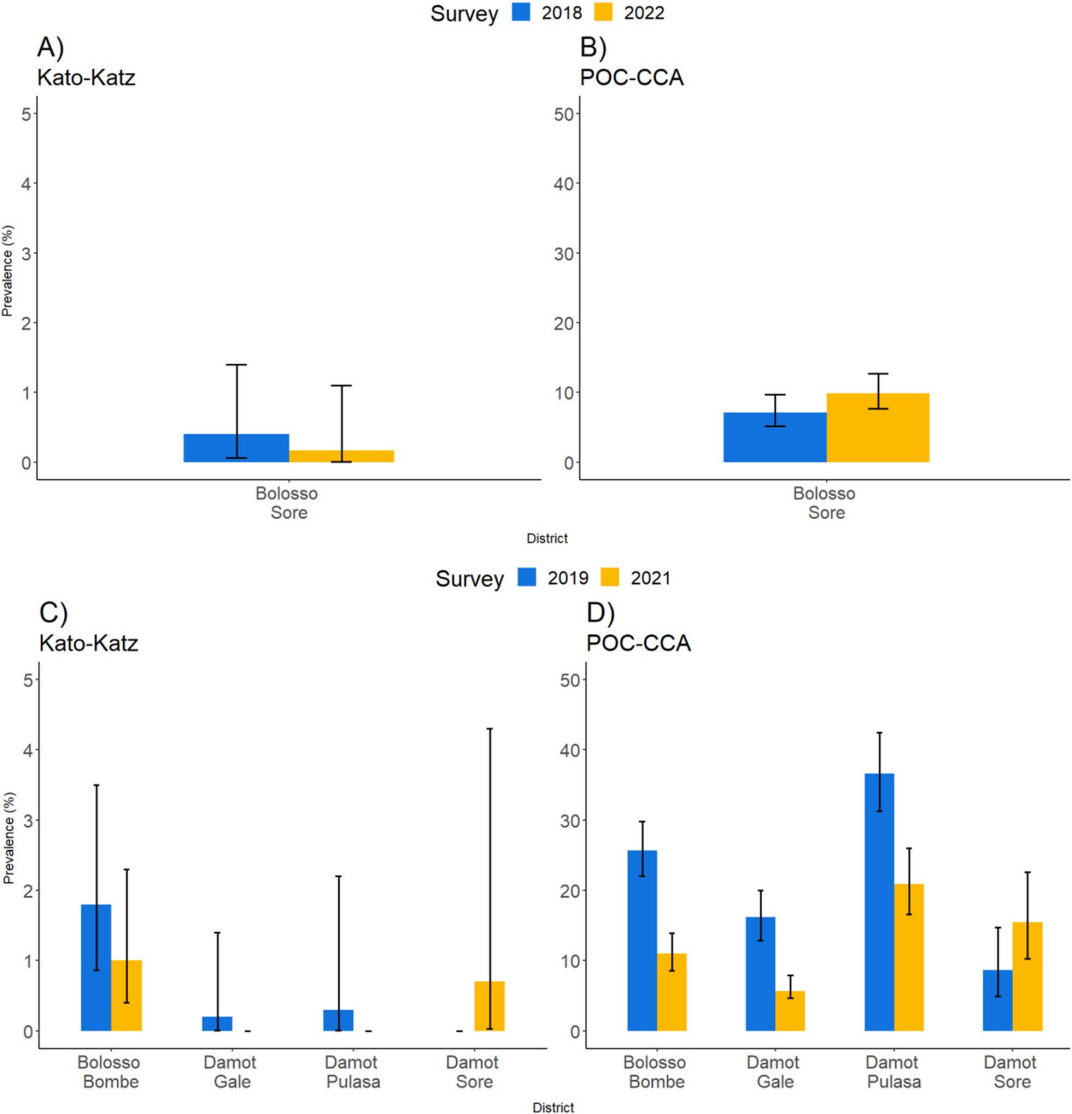

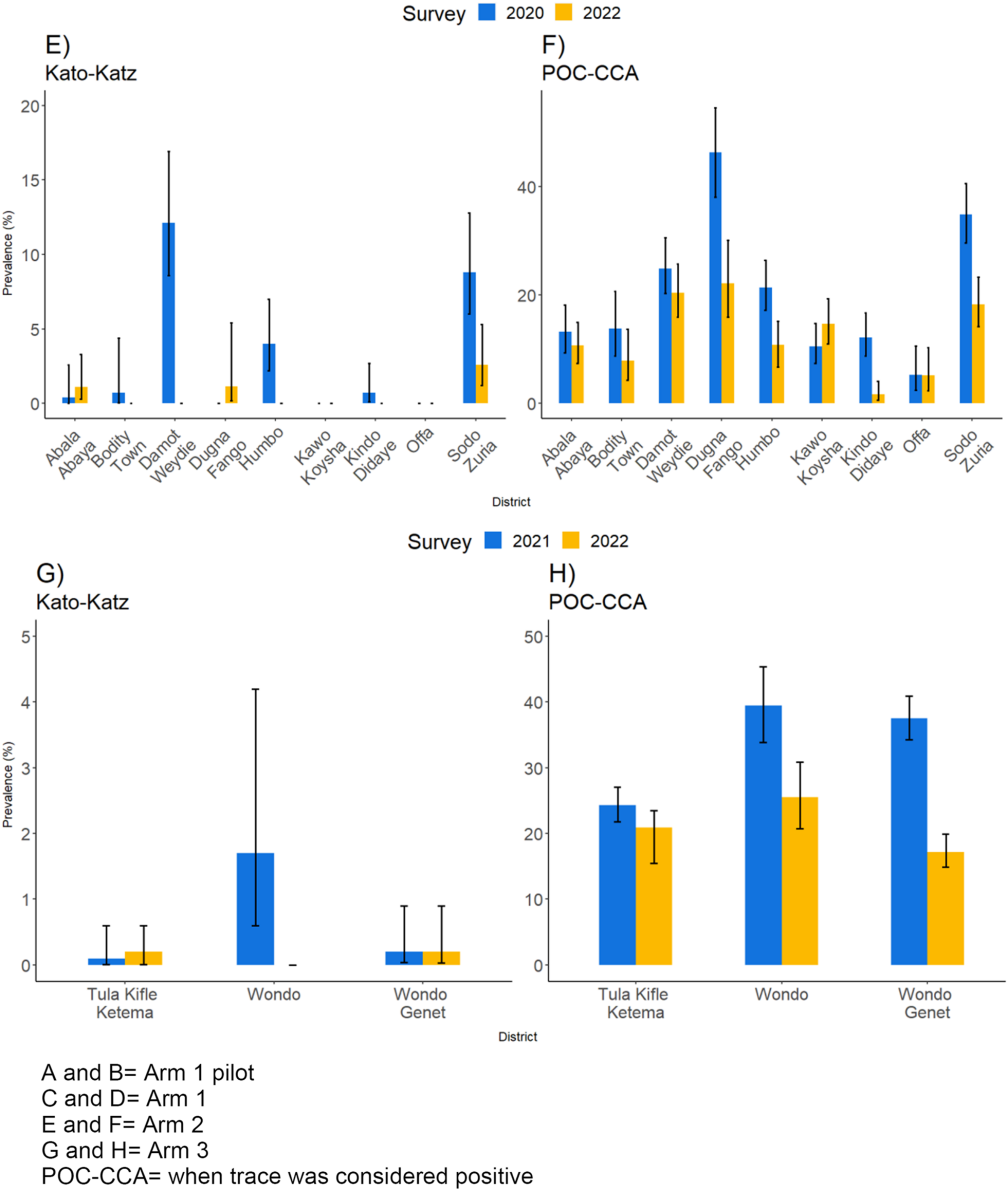
Trends in the prevalence of schistosome infection (*S. mansoni*) at the district level over the various cohort surveys. **A** and **B** Prevalence of SCH in Arm 1 pilot by KK and POC-CCA(+) at BL (2018) and FU3 (2022); **C** and **D** prevalence of SCH in Arm 1 by KK and POC-CCA(+) at BL (2019) and FU2 (2021); **E** and **F** prevalence of SCH in Arm 2 by KK and POC-CCA(+) at BL (2029) and FU2 (2021); **G** and **H** prevalence of SCH in Arm 3 by KK and POC-CCA(+) at BL (2021) and FU1 (2022). *BL* baseline

Figure 2 shows the heterogeneity in infection levels across districts and arms in the study site. In Arm 1, SCH prevalence was below 2% by KK but increased from baseline to FU3 by POC-CCA (7.1% vs. 9.9%).

*Schistosoma mansoni* infection showed a clear declining trend in all the districts and arms except in Damot Sore district (from 0% in 2020 to 0.7% in 2022) in Arm 1 and Diguna Fango district (from 0% in 2020 to 1.1% in 2022) in Arm 2. Fourteen districts (82%) recorded a prevalence of less than 1% by KK.

By POC-CCA, Damot Sore district in Arm 1 (from 8.7 to 15.5%) and Kawo Koysha district in Arm 2 (from 10.5% in 2020 to 14.7% in 2022) demonstrated increasing prevalence values, when trace was considered positive. In the remaining 15 districts (88%) of Arm 2, the prevalence of SCH decreased. The prevalence exhibited variability across districts, ranging from 1.7% to 25.7% in Kindo Didaye (in Arm 2) to Wondo district (in Arm 3) (Additional file 3: Table S3).

## Discussion

The prevalence of *S. mansoni* infections in the study area remained very low in all the arms of the Geshiyaro study area, ranging in levels from 0% to 2.6% using KK diagnostics. However, the prevalence was much higher as measured by the POC-CCA diagnostic (ranging 1.7–25.5% in the follow-up surveys), and heterogeneity in infection level across districts and arms was observed, when trace was considered either positive or negative. This is consistent with the findings of other published studies, such as school surveys in Burundi, Rwanda, and Egypt, where the national target of 2% was recorded based on KK, but when POC-CCA was used a very high prevalence (10–50%) [24] was found. A similar pattern has been recorded in other country locations (Hussien et al., in press]. In the present study in Southern Ethiopia, the utilization of the POC-CCA diagnostic indicates the presence of considerable *S. mansoni* transmission in all the study arms. According to the WHO reference guidance, transmission can be classified as low, medium, or high. The POC-CCA diagnostic identified 5–25%, 10–35%, and 15–40% for infection status levels of low, medium, and high; while using the KK diagnostic they were 1%, 5%, and 10% by KK duplicates, respectively [4].

In all the arms, the prevalence of *S. mansoni* as measured by the KK diagnostic remained low at baseline and follow-up survey. However, Arm 2 showed a significant change in prevalence over time (*t* = 6.89, *P* < 0.0001). On the other hand, when the POC-CCA test was employed, considering trace results as positive, a significant reduction in infection prevalence was observed in Arm 1, Arm 2, and Arm 3, but not in Arm 1 pilot. The limited reduction in Arm 1 pilot may be attributed to its status as the district where the project was initiated before expanding its focus to the other arms. In the Arm 1 pilot district (Boloso Sore), the implementation of MDA faced challenges due to resistance in the target population regarding the use of biometrics (fingerprints) for enrollment, as documented elsewhere (Birhan et. al, 2023, in press). When trace was considered negative, a significant decrease in prevalence was observed in Arm 1 and Arm 2.

A significant reduction in *S. mansoni* infection was found in Arm 1 and Arm 2, whether considering POC-CCA trace as positive or negative. These two arms seem to have benefited from the expanded community-wide treatment, encompassing all adults in the districts, in contrast to the school-based treatment only. The implementation of community-wide MDA is highly likely to influence transmission of SCH, even in areas characterized by low transmission [25]. In Arm 3, the reduction was significant only when considering the trace as positive.

The reduction in trace *S. mansoni* as diagnosed by POC-CCA was more pronounced in Arm 1 and Arm 3, potentially attributable to the high prevalence in these arms at baseline. Arm 1 and Arm 2 (except for trace) exhibited a consistent decline in all intensity categories (trace, 1+, 2+, and 3+). In contrast, Arm pilot displayed an increasing trend except for 3+, which declined. The fluctuation in trace levels underscores the potential individual variations in interpreting *S. mansoni* infection dynamics.

There was no notable difference in infection based on gender across arms, whether KK or POC-CCA was employed. However, with trace considered positive in POC-CCA, a higher rate of infection was observed in the 5–14-year age group (SAC) relative to the other age groups, which may have been due to contact with infected water, as is typically recorded in epidemiological studies of schistosome infections when stratified by age, with peaks in children [26].

The prevalence of urinary SCH was extremely low throughout the surveys, in line with the national mapping conducted in Ethiopia [13].

The evaluation of intervention groups (Arm 1 pilot, Arm 1, and Arm 2) against the control group (Arm 3) suggested that Arm 2 demonstrated effectiveness in reducing infection prevalence as measured by POC-CCA in the mid-term analysis. This finding may be influenced by the evolving intensive WaSH interventions and utilization in Arm 1 over time.

Arm 1 pilot showed an initial increase in prevalence during follow-up surveys (FU1 and FU2), then decreased to baseline levels by KK and CCA in FU3. Arm 1 and Arm 2 showed varying trends over follow-up surveys, while Arm 2 exhibited a decreasing trend in infection prevalence. This was attributed to variations in the treatment period, low coverage, occasional drug shortages, and impact of the COVID-19 pandemic.

In the majority of participants, the intensity of infection assessed by KK was categorized as light in all arms at the baseline, with notable reduction observed in Arm 2 during the latest follow-up. While there was a decrease in Arm 2, no significant change was observed in the other arms. Overall, the average egg count remained consistently low across the study. This could have resulted from the long-term deworming program even before the current project or the potentially low sensitivity of KK in detecting eggs in areas with low transmission [27].

In this study, the fact that transmission was much more substantial when measured by POC-CCA raises concerns. Detailed studies are needed to compare different schistosome infection diagnostics in low-, medium-, and high-transmission settings, considering the substantial variability in egg counts among recorded infections.

Damot Sore (Arm 1) and Kawo Koysha (Arm 2), along with all other districts in Arm 3, exhibited a higher risk of transmission when assessed using POC-CCA, regardless of whether trace results were considered positive or negative. These areas could potentially be hotspot districts. Nevertheless, a more comprehensive investigation is essential at the subdistrict level to precisely identify locations with high transmission rates. The observed heterogeneity in transmission across study districts underscores the focal nature of SCH infection. Targeted treatment in identified hotspots may be a practical approach for schistosome infection control, potentially reducing drug-related costs. However, there is an urgent need for evaluation, including understanding POC-CCA measures and assessing standards of manufacturing quality for the new diagnostic methods such as point-of-care circulating anodic antigen (POC-CAA) and quantitative polymerase chain reaction (qPCR) supplied by different manufacturers. This should be combined with careful evaluation of comparative values with KK scores, given that this diagnostic is cheap and simple to use and is the dominant diagnostic used in endemic areas. Such an evaluation needs to be supported by WHO and the major research funders in this area of tropical NTD control. The Geshiyaro sentinel sites would be a good location to conduct such comparative evaluations [17].

The low levels of infection recorded in the present study limit trend analyses of changes in the mean intensity of infection due to the small sample of infected individuals. Additionally, the comparison of different arms was influenced by variations in the implementation period.

## Conclusions

The study in general revealed a significant reduction in *S. mansoni* prevalence. However, variations in trends were observed across arms, age groups, and districts, highlighting the importance of considering different factors in SCH control programs. Moreover, elevated POC-CCA values may suggest continued transmission. There is a pressing need for a critical scientific review and evaluation of the performance of different diagnostic tests in detecting the presence and severity of schistosome infections. The observed spatial heterogeneity of infection in Southern Ethiopia calls for more localized disease monitoring and increased targeting of interventions at potential transmission hotspots.

### Supplementary Information

**Additional file 1. Table S1.** Number of sentinel site participants in different arms by age group and gender.

**Additional file 2. Table S2.** Reduction of SCH prevalence across all arms using POC-CCA (including trace, 1+, 2+, 3+ results).

**Additional file 3. Table S3.** Reduction of SCH prevalence by district.

## Data Availability

All data analyzed in this study are included in this published article.

## Abbreviations

BCC: Behavioral change communication
BL: Baseline
CI: Confidence interval
DALY: Disability-adjusted life years
EPG: Eggs per gram
FU: Follow-up
KK: Kato-Katz
MDA: Mass drug administration
NTD: Neglected tropical disease
POC-CAA: Point-of-care circulating anodic antigen
POC-CCA(−): Point-of-care circulating cathodic antigen when trace considered as negative
POC-CCA(+): Point-of-care circulating cathodic antigen when trace considered as positive
POC-CCA: Point-of-care circulating cathodic antigen
PZQ: Praziquantel
qPCR: Quantitative polymerase chain reaction
SAC: School-age children
SCH: Schistosomiasis
SH: *Schistosoma haematobium*
SM: *Schistosoma mansoni*
SNNPR: Southern Nations, Nationalities, and Peoples’ Region
STH: Soil-transmitted helminths
WaSH: Water, sanitation, and hygiene
WHO: World Health Organization
