# Correction: Progress in controlling the transmission of schistosome parasites in Southern Ethiopia: the Geshiyaro Project in the Wolaita Zone

**DOI:** 10.1186/s13071-024-06251-3

**Published:** 2024-03-28

**Authors:** Birhan Mengistu, Ewnetu Firdawek Liyew, Melkie Chernet, Geremew Tasew, Santiago Rayment Gomez, Rosie Maddren, Benjamin Collyer, Ufaysa Anjulo, Adugna Tamiru, Kathryn Forbes, Zelalem Mehari, Kebede Deribe, Teshale Yadeta, Mihretab Salasibew, Getachew Tollera, Roy Anderson

**Affiliations:** 1 London Centre for Neglected Tropical Disease Research, Department of Infectious Disease Epidemiology, Faculty of Medicine, St Marys Campus, Imperial College London, London, UK; 2 Bacterial, Parasitic and Zoonotic Disease Research Directorate, Ethiopian Public Health Institute, Addis Ababa, Ethiopia; 3 Disease Prevention and Health Promotion Core Process, Ministry of Health, Addis Ababa, Ethiopia; 4 Children’s Investment Fund Foundation, London, UK

**Correction: Parasites & Vectors (2024) 17:113** 10.1186/s13071-024-06156-1

Following publication of the article [1], the authors flagged that the family name of the first author had been incorrectly spelled as 'Mengsitu' instead of 'Mengistu'. The name has since been corrected in the article. The authors thank you for reading and apologize for any inconvenience caused.

## References

[1] Mengistu B, Liyew EF, Chernet M, Tasew G, Gomez SR, Maddren R, Collyer B, Anjulo U, Tamiru A, Forbes K, Mehari Z, Deribe K, Yadeta T, Salasibew M, Tollera G, Anderson R (2024). Progress in controlling the transmission of schistosome parasites in Southern Ethiopia: the Geshiyaro Project in the Wolaita Zone. Parasites Vectors.

