# Supplementary material for: Progress in controlling the transmission of schistosome parasites in Southern Ethiopia: the Geshiyaro Project in the Wolaita Zone
Source: Parasit Vectors. 2024 Mar 6;17:113. doi: 10.1186/s13071-024-06156-1 (PMC10919034; doi:10.1186/s13071-024-06156-1)
Supplement: Supplementary file 1 — Additional file 1. Table S1. Number of sentinel site participants in different arms by age group and gender. [file 13071_2024_6156_MOESM1_ESM.docx]

**Additional file 1: Table S1.** Number of sentinel site participants in different arms by age group and gender.

|  |  | Baseline | FU1 | FU2 | FU3 |
| --- | --- | --- | --- | --- | --- |
| Arm 1 pilot district | Age group (Years) |  |  |  |  |
|  | 2-4 | 33 | 95 | 55 | 91 |
|  | 5-14 | 193 | 153 | 175 | 163 |
|  | 15-19 | 55 | 72 | 49 | 50 |
|  | 20-35 | 148 | 145 | 110 | 127 |
|  | 35+ | 139 | 122 | 164 | 153 |
|  | Gender |  |  |  |  |
|  | Male | 325 | 324 | 297 | 325 |
|  | Female | 243 | 263 | 256 | 259 |
|  | Total | 568 | 587 | 553 | 584 |
|  | Available for KK analysis | 568 | 587 | 553 | 584 |
|  | Available for POC-CCA analysis | 547 | 579 | 552 | 584 |
|  | Available for dipstick test | 547 | 579 | 552 | 581 |
|  | Available for urine filtration | 0 | 0 | 0 | 7 |
| Arm 1 | Age group (Years) |  |  |  |  |
|  | 2-4 | 258 | 292 | 233 |  |
|  | 5-14 | 359 | 375 | 469 |  |
|  | 15-19 | 191 | 250 | 201 |  |
|  | 20-35 | 302 | 343 | 350 |  |
|  | 35+ | 285 | 338 | 384 |  |
|  | Sex |  |  |  |  |
|  | Male | 713 | 802 | 829 |  |
|  | Female | 682 | 796 | 806 |  |
|  | Total | 1395 | 1598 | 1636 |  |
|  | Available for KK analysis | 1395 | 1598 | 1636 |  |
|  | Available for POC-CCA analysis | 1390 | 1598 | 1636 |  |
|  | Available for dipstick test | 1390 | 1598 | 1634 |  |
|  | Available for urine filtration | 61 | 68 | 29 |  |
| Arm 2 | Age group (Years) |  |  |  |  |
|  | 2-4 | 398 | 342 | 297 |  |
|  | 5-14 | 512 | 551 | 644 |  |
|  | 15-19 | 400 | 359 | 352 |  |
|  | 20-35 | 443 | 433 | 442 |  |
|  | 35+ | 408 | 431 | 471 |  |
|  | Sex |  |  |  |  |
|  | Male | 1114 | 1089 | 1144 |  |
|  | Female | 1047 | 1024 | 1059 |  |
|  | Total | 2161 | 2113 | 2203 |  |
|  | Available for KK analysis | 2161 | 2113 | 2203 |  |
|  | Available for POC-CCA analysis | 2156 | 2113 | 2203 |  |
|  | Available for dipstick test | 2156 | 2113 | 2203 |  |
|  | Available for urine filtration | 87 | 58 | 37 |  |
| Arm 3 | Age group (Years) |  |  |  |  |
|  | 2-4 | 414 | 254 |  |  |
|  | 5-14 | 507 | 680 |  |  |
|  | 15-19 | 403 | 400 |  |  |
|  | 20-35 | 481 | 488 |  |  |
|  | 35+ | 372 | 416 |  |  |
|  | Sex |  |  |  |  |
|  | Male | 1107 | 1154 |  |  |
|  | Female | 1070 | 1084 |  |  |
|  | Total | 2177 | 2238 |  |  |
|  | Available for KK analysis | 2177 | 2238 |  |  |
|  | Available for POC-CCA analysis | 2156 | 2115 |  |  |
|  | Available for dipstick test | 2156 | 2115 |  |  |
|  | Available for urine filtration | 125 | 82 |  |  |

BL= Baseline; FU= Follow-up

*In Arm 1 pilot: FU1 was in 2019, FU2 was in 2020, FU3 was in 2021, and FU4 was in 2022

*In Arm 1 and Arm 2: FU1 was in 2020, FU2 was in 2021, and FU2 was in 2022

*In Arm 3: FU1 was in 2021, and FU2 was in in 2022
