# Supplementary material for: Progress in controlling the transmission of schistosome parasites in Southern Ethiopia: the Geshiyaro Project in the Wolaita Zone
Source: Parasit Vectors. 2024 Mar 6;17:113. doi: 10.1186/s13071-024-06156-1 (PMC10919034; doi:10.1186/s13071-024-06156-1)
Supplement: Supplementary file 2 — Additional file 2. Table S2. Reduction of SCH prevalence across all arms using POC-CCA (including trace, 1+, 2+, 3+ results). [file 13071_2024_6156_MOESM2_ESM.docx]

**Additional file 2: Table S2.** Reduction of SCH prevalence across all arms using POC-CCA (including trace, 1+, 2+, 3+ result)

| Arm | Survey | Negative (% and 95%CI) | Trace (% and 95% CI) | 1+ (% and 95% CI) | 2+ (% and 95% CI) | 3+ (% and 95% CI) |
| --- | --- | --- | --- | --- | --- | --- |
| Arm 1 pilot | Baseline | 92.9 (90.3, 92.9) | 4.4 (2.9, 6.6) | 1.3 (0.6, 2.7) | 0.7(0.2, 2) | 0.7(0.2, 2) |
|  | FU1 | 83.1 (81.9-87.9) | 5.7 (4. 8) | 5.3 (3.7, 7.6) | 2.9 (1.8, 4.8) | 0.9 (0.3, 2.1) |
|  | FU2 | 85.5 (82.2, 88.3) | 8 (5.9, 10.6) | 5.3 (3.6, 7.5) | 0.9 (0.3, 2.2) | 0.4 (0.1, 1.5) |
|  | FU3 | 90.1 (87.3, 92.3) | 6.5 (4.7, 8.9) | 1.5 (0.8, 3) | 0.9 (0.3, 2.1) | 1 (0.4, 2.3) |
| Arm 1 | Baseline | 76.8 (74.4, 78.9) | 11.9 (10.3, 13.8) | 5.5 (4.4, 6.9) | 4.2 (3.2, 5.4) | 1.6 (1, 2.4) |
|  | FU1 | 81.9 (79.9, 83.7) | 12.7 (11.1, 14.5) | 2.2 (1.6, 3.1) | 2.1 (1.5, 3) | 1.1 (0.7, 1.8) |
|  | FU2 | 87.1 (85.4, 88.6) | 3.5 (2.6, 4.4) | 5 (4.1, 6.2) | 2 (1.4, 2.9) | 0.6 (0.3, 1.1) |
| Arm 2 | Baseline | 79.7 (78, 81.4) | 4.7 (3.8, 5.7) | 6.1 (5.2, 7.2) | 5.2 (4.4, 6.3) | 4.2 (3.4, 5.2) |
|  | FU1 | 87.4 (85.9, 88.8) | 5.6 (4.7, 6.7) | 3.1 (2.4, 4) | 2.6 (2, 3.4) | 1.2 (0.8, 1.8) |
|  | FU2 | 87.5 (86.1, 88.9) | 4.4 (3.6, 5.3) | 4.8 (4, 5.8) | 3.2 (2.5, 4) | 0.1 (0, 0.4) |
| Arm 3 | Baseline | 68.4 (66.4, 70.3) | 13.9 (12.5, 15.4) | 9.3 (8.1, 10.6) | 7.1 (6.1, 8.3) | 1.4 (0.9, 2) |
|  | FU1 | 79.9 (78.2, 81.6) | 4.5 (3.7, 5.5) | 7.9 (6.8, 9.1) | 5.8 (4.9, 6.8) | 1.9 (1.4, 2.6) |

*In Arm 1 pilot: FU1 was in 2019, FU2 was in 2020, FU3 was in 2021, and FU4 was in 2022

*In Arm 1 and Arm 2: FU1 was in 2020, FU2 was in 2021, and FU2 was in 2022

*In Arm 3: FU1 was in 2021, and FU2 was in in 2022
