# Supplementary material for: Progress in controlling the transmission of schistosome parasites in Southern Ethiopia: the Geshiyaro Project in the Wolaita Zone
Source: Parasit Vectors. 2024 Mar 6;17:113. doi: 10.1186/s13071-024-06156-1 (PMC10919034; doi:10.1186/s13071-024-06156-1)
Supplement: Supplementary file 3 — Additional file 3. Table S3. Reduction of SCH prevalence by district. [file 13071_2024_6156_MOESM3_ESM.docx]

**Additional file 3: Table S3.** Reduction of SCH prevalence by district

| Arm | District | Baseline prevalence% (95%CI) | Follow up prevalence% (95%CI) | Baseline prevalence% (95%CI) | Follow up prevalence% (95%CI) | Baseline prevalence% (95%CI) | Follow up prevalence% (95%CI) |
| --- | --- | --- | --- | --- | --- | --- | --- |
|  |  | KK | | CCA+ | | CCA- | |
| Arm 1 (BS) | Bolosso Sore | 0.4(0.06,1.4) | 0.17(0.008,1.1) | 7.1(5.2,9.7) | 9.9(7.7,12.7) | 2.7(1.6,4.6) | 3.4(2.5,5.3) |
| Arm 1 (FD) | Boloso Bombe | 1.8(0.87.3.5) | 1(0.4,2.3) | 25.7(22,29.8) | 11(8.6,13.9) | 13.1(10.3,16.4) | 7.2(5.3,9.7) |
|  | Damot Gale | 0.2(0.01,1.4) | 0 | 16.2(12.9,20) | 5.7(4,7.9) | 10.1(7.5,13.4) | 4.7(3.2,6.7) |
|  | Damot Pulasa | 0.3(0.01,2.2) | 0 | 36.6(31.2,42.5) | 20.9(16.6,26) | 13.2(9.7,17.8) | 12.7(9.3,17.1) |
|  | Damot Sore | 0 | 0.7(0.03,4.3) | 8.7(4.9,14.7) | 15.5(10.3,22.6) | 4.7(2.1,9.7) | 12.8(8.1,19.6) |
| Arm 2 | Abala Abaya | 0.4(0.02,2.6) | 1.1(0.3,3.3) | 13.2(9.3,18.2) | 10.7(7.4,15) | 8.2(5.2,12.6) | 4.3(2.3,7.5) |
|  | Bodity Town | 0.7(0.04,4.4) | 0 | 13.8(8.8,20.7) | 7.9(4.3,13.7) | 8.2(5.2,12.6) | 5.3(2.5,10.5) |
|  | Damot Weydie | 12.1(8.6,16.9) | 0 | 24.9(20.30.6) | 20.4(15.9,25.7) | 7.6(4,13.5) | 20(15.5,25.3) |
|  | Diguna Fango | 0 | 1.14(0.2,5.4) | 46.3(38.1,54.6) | 22.2(15.9,30.1) | 48.2(38.1,54.6) | 13.2(8.3,20.1) |
|  | Humbo | 4(2.2,7) | 0 | 21.4(17.2,26.4) | 10.8(76.7,15.2) | 18.6(14.6,23.4) | 4.4(2.5,7.6) |
|  | Kawo Koysha | 0 | 0 | 10.5(7.4,14.8) | 14.7(11,19.3) | 4.1(2.2,7.2) | 14.7(11,19.3) |
|  | Kindo Didaye | 0.7(0.1,2.7) | 0 | 12.2(8.8,16.7) | 1.7(0.6,4.1) | 7.7(5,11.6) | 0.3(0.01,2.1) |
|  | Offa | 0 | 0 | 5.3(2.5,10.6) | 5.2(2.4,10.3) | 0 | 0.6(0.03,4) |
|  | Sodo Zuria | 8.8(6,12.8) | 2.6(1.2,5.3\|) | 34.9(29.6,40.6) | 18.3(14.2,23.3) | 28.8(23.8,34.2) | 8.3(5.5,12.2) |
| Arm 3 | Tula Kifle Ketema | 0.1(0.005,0.6 | 0.1(0.005,0.6 | 24.3(21.8,27.1) | 20.9(15.5,23.5) | 15.2(13.1,17.6) | 15.3(13.2,17.6) |
|  | Wondo | 1.7(0.6,4.2) | 0 | 39.5(33.9,45.4) | 25.5(20.7,30.9) | 26.5(21.6,32) | 18.5(14.3,23.4) |
|  | Wondo Genet | 0.2(0.04,0.9) | 0.2(0.03,0.9) | 37.5(34.3,40.9) | 17.2(14.9,19.9) | 17.8(15.3,20.5) | 14.9(12.7,17.4) |

- In Arm 1 pilot: baseline was done in 2019 and follow up in 2022
- In Arm 1: baseline was done in 2020 and follow up in 2022
- In Arm 2: baseline was done in 2020 and follow up in 2022
- In Arm 3: baseline was done in 2021 and follow up in 2022
